# High **α**-SMA expression in the tumor stroma is associated with adverse clinical parameters in mismatch repair–proficient colorectal cancers only

**DOI:** 10.1093/ajcp/aqae145

**Published:** 2024-11-04

**Authors:** Declan J Sculthorpe, Amy Denton, Wakkas Fadhil, Dewi Rusnita, Mohammad Ilyas, Abhik Mukherjee

**Affiliations:** Molecular Pathology Research, Biodiscovery Institute, School of Medicine, University of Nottingham, Nottingham, UK; Molecular Pathology Research, Biodiscovery Institute, School of Medicine, University of Nottingham, Nottingham, UK; Molecular Pathology Research, Biodiscovery Institute, School of Medicine, University of Nottingham, Nottingham, UK; Molecular Pathology Research, Biodiscovery Institute, School of Medicine, University of Nottingham, Nottingham, UK; Molecular Pathology Research, Biodiscovery Institute, School of Medicine, University of Nottingham, Nottingham, UK; Department of Histopathology, Nottingham University Hospitals NHS Trust, Queen’s Medical Centre, Nottingham, UK; Molecular Pathology Research, Biodiscovery Institute, School of Medicine, University of Nottingham, Nottingham, UK; Department of Histopathology, Nottingham University Hospitals NHS Trust, Queen’s Medical Centre, Nottingham, UK

**Keywords:** colorectal cancer, biomarker analysis, immunohistochemistry, DNA mismatch repair, α-SMA, digital image analysis

## Abstract

**Objectives:**

As mismatch repair status confers differential prognosis in colorectal cancers, this study aimed to determine associations of α–smooth muscle actin (α-SMA) protein expression in mismatch repair–proficient (pMMR) and mismatch repair–deficient (dMMR) colorectal tumors with clinicopathologic and prognostic features.

**Methods:**

Tissue microarrays from patients with colorectal cancer, immunostained with α-SMA, were assessed through digital image analysis. Total (n = 962), pMMR (n = 782), and dMMR (n = 156) stromal H-scores were assessed for associations with clinicopathologic and survival data.

**Results:**

Higher α-SMA expression was correlated with pMMR status (*P* = 5.2223 × 10^–8^). In the pMMR subgroup, higher α-SMA stromal expression at the tumor periphery was correlated with higher T stage (*P* = .002), perineural invasion (*P* = .038), infiltrative tumor edge (*P* = .01), involved nodal status (*P* = .036), metastases (*P* = .013), synchronous metastases (*P* = .007), recurrence (*P* = .004), and both 3-year and 5-year survival (*P* = .018). dMMR tumors showed no significant correlations with α-SMA staining.

**Conclusions:**

The findings highlight that immunostaining with α-SMA in pMMR colorectal tumors, especially at the tumor periphery, has the potential to identify patients with adverse prognostic features. Digital assessment of α-SMA may offer improved objectivity, accuracy, economy of time, and risk stratification for management.

**Key Points**

- Higher α–smooth muscle actin (α-SMA) expression, especially at the tumor periphery, in mismatch repair–proficient colorectal cancers is associated with poorer prognostic clinicopathologic variables.
- The α-SMA staining in mismatch repair–proficient tumors may be used clinically to indicate propensity of locoregional spread and metastases.
- Digital assessment may overcome the challenges in manually assessing stromal immunostains in colorectal cancers and may help to improve objectivity, increase accuracy, and economize pathologists’ time.

## INTRODUCTION

Metastases and recurrence are common clinical complications of colorectal cancer (CRC), the fourth most common cancer in the United Kingdom and globally the third most common cancer.^1,2^ Epithelial-mesenchymal transition (EMT) is one of the processes that facilitates such metastases, whereby stationary polarized epithelial cells acquire mesenchymal characteristics through multiple molecular and morphologic changes.^3,4^ As the molecular profiling of CRC becomes delineated through the 4 consensus molecular subtypes (CMSs), CMS4, the mesenchymal subtype, has been shown to have the poorest prognosis.^5^ These are generally tumors with activated stromal remodeling pathways and are usually proficient for DNA mismatch repair (pMMR) systems. In contrast, the deficient mismatch repair (dMMR) tumors cluster to the relatively favorable prognostic CMS1, unless there is recurrence.^6^ CMS2 (mostly pMMR) and CMS3 (mixed mismatch repair [MMR] status) are also of favorable prognosis compared to CMS4 and do not show the mesenchymal activation characteristics of CMS4.^5-8^ These profiles not only indicate a link between MMR status and prognosis but also show the heterogeneity among pMMR tumors in terms of outcome. Therefore, simple stains, easily applicable on clinical samples, are needed to determine the contribution of the stromal compartment to CRC prognostication within the context of common clinically assessed molecular subtypes by MMR status.

One of the common molecules linked to EMT and tumor stromal remodeling is α–smooth muscle actin (α-SMA), an isoform of actin encoded by ACTA2 and expressed in smooth muscle cells, myofibroblasts, blood vessels,^9,10^ and cancer-associated fibroblasts (CAFs).^11^ Studies to date in CRC regarding α-SMA have revealed divergent results, with 1 study demonstrating that greater α-SMA positivity within the stromal area has been found associated with shorter disease-free survival but not with clinicopathologic variables.^12^ On the other hand, another study indicates that α-SMA (high) but podoplanin-negative CAFs were associated with adverse clinicopathologic parameters and tend to exhibit shorter disease-free survival time, although this does not reach statistical significance.^11^ The role of α-SMA in tumoral stroma in MMR-proficient vs MMR-deficient tumors has not been studied, and their links to clinicopathologic parameters and prognosis within the context of this clinically commonly used molecular subtyping of CRCs have not been explored previously. This led us to explore the stromal expression of α-SMA in pMMR vs dMMR CRCs and their correlations with clinicopathologic variables and prognosis. In comparison to epithelial stains, as scoring a stromal stain like α-SMA is morphologically complex to be undertaken manually, a digital-based analysis was performed.

## MATERIALS AND METHODS

### Tissue Microarray Construction and Staining

Tissue microarrays (TMAs) of CRC samples (n = 1000) from a tertiary institution, with cases ranging from July 2008 to September 2014, were arrayed per standard protocols (ethical reference: REC reference: 23/EM/0079). Four tissue cores were included for each case: 3 tumor luminal, central, and peripheral compartments (to account for variability and heterogeneity from the mucosal to the pericolic plane) and 1 from adjacent normal.

Slides were stained with clinical grade α-SMA antibody (Roche, prediluted supply) by using the BenchMark ULTRA System (Roche) per protocol. Slides were digitized using the Ventana DP200 slide scanner (Roche) at ×40 using routine settings, and images were saved as TIF files. The MMR status was determined as per standard pathology practice using clinical grade MMR markers (MLH1, PMS2, MSH2, and MSH6; Roche, prediluted supply) by using the BenchMark ULTRA System (Roche) per protocol.

### Automated Evaluation of α-SMA Staining

**FIGURE 1:**
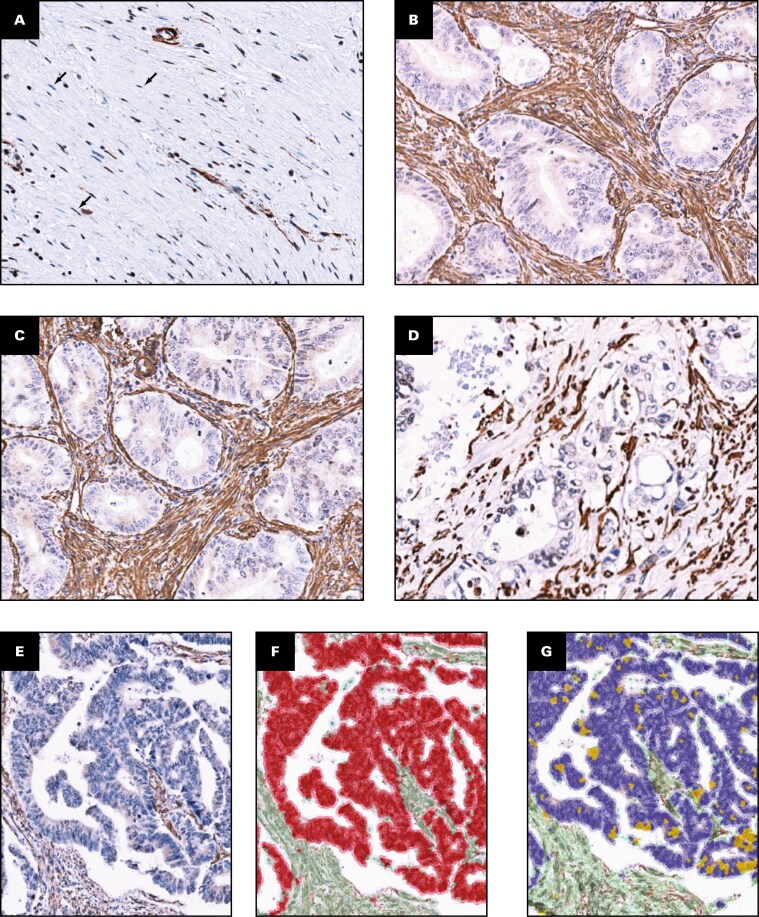
Examples of staining within the stroma for α-SMA and automated classifier development for α-SMA on QuPath. **A**, Stroma that is mainly negative for α-SMA (indicated by arrows). **B**, Stroma that is strongly positive for α-SMA. **C**, **D**, Examples of the 2 different staining intensities (1+/2+) for α-SMA within the stroma. **E**, Colorectal cancer TMA core with no segmentation or intensity classification applied. **F**, Cell segmentation classifier applied, tumor epithelial cells in red and stromal cells in green. **G**, Stain intensity classification applied, negative tumor epithelial cells in blue and positive tumor epithelial cells in yellow (not assessed in this study). Negative stromal cells in light green, positive stromal cells in dark green. α-SMA, α–smooth muscle actin; TMA, tissue microarray.

QuPath 0.4.0^13^ was used to generate automated stromal H-scores for α-SMA. A project was created for α-SMA, using File → Project → Create new project. The 15 TMA slide images were imported into the project using File → Project → Add Images. All images were set as Brightfield H-DAB. The first slide in the TMA series was used as the index slide to detect tissue and cells. Using the “wand tool” detected stromal cells, and tumor epithelial cells were annotated for compartment classification; blood vessels and muscle that stained positively for SMA were excluded. Annotations were supervised by consultant histopathologist (AM) input. Compartments were then segmented using QuPath’s segmentation tool. Cell intensity classification was then performed to set thresholds for the staining intensities FIGURE 1. The intensity thresholds were determined by calculating a mean from the cell intensity of individual stromal cells within at least 10 randomly selected cores over at least 3 randomly selected TMA slides within the project that had been manually assessed by a histopathologist (AM) to be at that intensity. Intensities of 0, 1+, or 2+ scores were deemed negative, weak, or strong, respectively, and automated α-SMA stromal H-scores (range, 0-200) were produced for each slide. The average scores were calculated for the stromal H-score by determining an average from available luminal, central, and peripheral scores in each case. Individual tumor compartments were also assessed.

### MMR Status Assessment

MMR status was defined as proficient (presence of nuclear staining of all 4 MMR proteins) or deficient (absent nuclear stain for 1 or more of the 4 MMR proteins), as per standard clinical practice.

### Statistical Analysis

Of the 1000 CRCs, 962 were available for assessment of α-SMA. A total of 782 pMMR and 156 dMMR confirmed cases were available for further subgroup analyses. To dichotomize the average α-SMA stromal H-scores into low and high, the median cutoff was selected. IBM SPSS Statistics 28 was used to assess clinicopathologic correlations. Tumor vs adjacent normal was compared through paired *t* test analyses and correlations generated through χ^2^ analyses. The strength of significant associations, allowing for multiparameter analyses, was tested through adjusted residuals (±2 taken to be representative of significant association). Kaplan-Meier followed by Cox regression for multivariate analyses was employed to determine survival associations.

## RESULTS

### Cohort Characteristics

The total cohort included 432 female and 568 male patients with a mean age of 68.81 years (range, 16-94 years) and is representative in terms of distribution of clinicopathologic features (Supplementary Table 1; all supplementary material is available at *American Journal of Clinical Pathology* online). The median survival was 112 months. The pMMR data set also maintained the distribution of clinicopathologic variables (329 female and 453 male patients, with a mean age of 68.33 years). The dMMR data set contained nearly equal proportions of male and female patients (74 female and 79 male patients, with a mean age of 71.46 years).

### Complete Cohort, Clinicopathologic Correlations, and Survival Analyses

Automated analyses of immunostain for the total cohort were completed in a span of 1 week. The average tumor-stromal α-SMA H-scores had a mean greater than the normal stromal α-SMA H-score (*P* < .001).

In the total cohort, higher α-SMA stromal expression was correlated with left-sided tumors (*P* = .01), higher T stages (*P* = .000324), vascular invasion (*P* = .047), perineural invasion (*P* = .003), infiltrative tumor edge pattern (*P* = 2.8301 × 10^–8^), higher N stages (*P* = .022), involved nodal status (*P* = .007), disease recurrence (*P* = .000156), metastasis (*P* = .005), synchronous metastasis (*P* = .005), and proficient MMR status (*P* = 5.2223 × 10^–8^) (Supplementary Table 2). When assessed individually in the tumor compartments, higher α-SMA stromal H-scores were associated with grade 2 tumors (*P* = .038), the infiltrative tumor edge pattern (*P* = .002), and proficient MMR status (*P* = .000047) in all tumor compartments. Lower α-SMA stromal expression was also correlated with lower T stages (T1/T2) in all tumoral compartments (*P* = .02). Higher luminal α-SMA stromal H-scores were associated with increased disease recurrence (*P* = .000169), left-sided tumors (*P* = .002), N1 stage (*P* = .022), and vascular invasion (*P* = .043) (Supplementary Table 3). Higher central α-SMA stromal H-scores were associated with N1 stage (*P* = .029), involved nodal status (*P* = .011), and a conspicuous peritumoral lymphocytic infiltrate (*P* = .020) (Supplementary Table 4). Higher peripheral α-SMA stroma H-scores were associated with disease recurrence (*P* = .003), metastasis (*P* = .000473), synchronous metastasis (*P* = .00021), perineural invasion (*P* = .008), and poorer 3-year (*P* = .013) and 5-year survival status (*P* = .045) (Supplementary Table 5).

**FIGURE 2:**
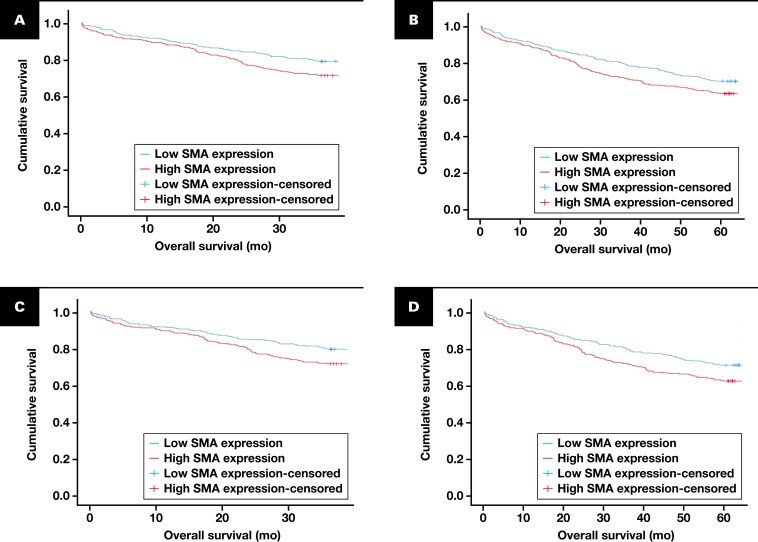
Kaplan-Meier survival analyses of automated α-SMA stromal H-scores in patients with colorectal cancer. **A**, A 3-year survival plot for automated α-SMA stromal expression at the tumor periphery within the total cohort (*P* = .013). **B**, A 5-year survival plot for automated α-SMA stromal expression at the tumor periphery within the total cohort (*P* = .033). **C**, A 3-year survival plot for automated α-SMA stromal expression at the tumor periphery within the mismatch repair proficient cohort (*P* = .019). **D**, A 5-year survival plot for automated α-SMA stromal expression at the tumor periphery within the mismatch repair–proficient cohort (*P* = .015) (*P* values from univariate analysis). α-SMA, α–smooth muscle actin.

Kaplan-Meier survival analysis was carried out for the average α-SMA H-score as well as each individual tumoral compartment stromal H-score. Significant associations between α-SMA expression and survival were found only at the peripheral tumor compartment. Higher α-SMA at the peripheral tumor compartment was associated with poorer 3-year (*P* = .013) and 5-year (*P* = .033) survival FIGURE 2A, B. Following multivariate analysis with the covariates of T stage, nodal stage, metastasis, and grade, high α-SMA expression at the peripheral compartment was not a significant independent prognostic indicator of 3-year (*P* = .097) or 5-year (*P* = .089) survival for the whole cohort.

### Proficient MMR Subgroup, Clinicopathologic Correlations, and Survival Analyses

Due to distinct differences in the pathophysiology of pMMR and dMMR tumors, as well as the significant correlations with pMMR status and high α-SMA stromal expression in the total cohort, subgroup analyses of pMMR/dMMR tumors were carried out. Higher α-SMA stromal H-scores in pMMR tumors were associated with similar poor prognostic variables, including higher T stage (*P* = .00005), vascular invasion (*P* = .02), perineural invasion (*P* = .021), infiltrative tumor edge pattern (*P* = 6.3029 × 10^–8^), and disease recurrence (*P* = .002) TABLE 1.

**TABLE 1:** χ^2^ Analysis for Associations of the pMMR Cohorts Average Stromal α-SMA H-Score With Clinicopathologic Variables^a^

Clinicopathologic features	pMMR average stromal α-SMA H-score		Adjusted residuals		χ² *P* value
	Low, No. (%)	High, No. (%)	Low	High	
Age					.12
Under 50	23 (41.8)	32 (58.2)	–1.6	1.6	
Over 50	383 (52.7)	344 (47.3)	1.6	–1.6	
3-year survival status					.329
Alive	316 (52.8)	283 (47.2)	1	–1	
Dead	88 (48.6)	93 (51.4)	–1	1	
5-year survival status					.819
Alive	276 (52.1)	254 (47.9)	0.2	–0.2	
Dead	128 (51.2)	122 (48.8)	–0.2	0.2	
Synchronous metastasis					.072
Metachronous	362 (53.2)	319 (46.8)	1.8	–1.8	
Synchronous	44 (43.6)	57 (56.4)	–1.8	1.8	
Disease recurrence					**.002**
No recurrence	301 (55.6)	240 (44.4)	3.1	–3.1	
Recurrence	105 (43.6)	136 (56.4)	–3.1	3.1	
Tumor site					0.077
Right colon	167 (54.9)	137 (45.1)	1.3	–1.3	
Left colon	148 (46.4)	171 (53.6)	–2.6	2.6	
Rectum	75 (56.8)	57 (43.2)	1.2	–1.2	
Transverse colon	16 (59.3)	11 (40.7)	0.8	–0.8	
T stage					**.000050**
T1	46 (70.8)	19 (29.2)	3.2	–3.2	
T2	61 (67.0)	30 (33.0)	3.1	–3.1	
T3	194 (49.0)	202 (51.0)	–1.7	1.7	
T4	105 (45.7)	125 (54.3)	–2.3	2.3	
Nodal stage					.27
N0	230 (53.3)	200 (46.7)	1.5	–1.5	
N1	92 (46.7)	105 (53.3)	–1.4	1.4	
N2	64 (49.6)	65 (50.4)	–0.4	0.4	
Nodal status					.125
Uninvolved	230 (53.3)	200 (46.7)	1.5	–1.5	
Involved	156 (47.9)	170 (52.1)	–1.5	1.5	
Metastasis					.09
Absent	362 (53.1)	320 (46.9)	1.7	–1.7	
Present	44 (44.0)	56 (56.0)	–1.7	1.7	
Tumor grade					.086
G1	7 (50.0)	7 (50.0)	–0.1	0.1	
G2	372 (51.1)	356 (48.9)	–1.8	1.8	
G3	27 (69.2)	12 (30.8)	2.2	–2.3	
Vascular invasion					**.022**
Absent	210 (55.7)	167 (44.3)	2.3	–2.3	
Present	185 (47.4)	205 (52.6)	–2.3	2.3	
Perineural invasion					**.021**
Absent	319 (53.3)	280 (46.7)	2.3	–2.3	
Present	65 (42.8)	87 (67.2)	–2.3	2.3	
Lymphovascular invasion					.950
Absent	250 (50.4)	246 (49.6)	–0.1	0.1	
Present	116 (50.7)	113 (49.3)	0.1	–0.1	
Tumor edge					**3.2145E-8**
Infiltrative	129 (39.0)	202 (61.0)	–5.5	5.5	
Pushing	157 (62.1)	96 (33.7)	5.5	–5.5	
Tumor budding					.094
Low	198 (51.4)	187 (48.6)	1.7	–1.7	
High	85 (44.0)	108 (56.0)	–1.7	1.7	
Peritumoral lymphocytes					.946
Inconspicuous	215 (49.3)	221 (50.7)	0.1	–0.1	
Conspicuous	73 (49.0)	76 (51.0)	–0.1	0.1	

α-SMA, α–smooth muscle actin; pMMR, mismatch repair proficient.

^a^Adjusted residuals designate correlation strength in multiparameter analysis; significant *P* values highlighted in bold.

Investigation of α-SMA stromal expression in individual tumor compartments revealed that higher peripheral α-SMA stroma H-scores were associated with T4 stage (*P* = .002), perineural invasion (*P* = .038), involved nodal status (*P* = .036), disease recurrence (*P* = .004), metastasis (*P* = .013), synchronous metastasis (*P* = .007), and poorer 3-year (*P* = .018) and 5-year survival status (*P* = .018) TABLE 2.

**TABLE 2:** Chi-Squared Analysis for Associations of the pMMR Cohorts Peripheral Stromal α-SMA H-Score With Clinicopathologic Variables^a^

Clinicopathologic features	pMMR peripheral stromal α-SMA H-score		Adjusted residuals		χ² *P* value
	Low, No. (%)	High, No. (%)	Low	High	
3-year survival status					**.018**
Alive	275 (54.8)	227 (45.2)	2.4	–2.4	
Dead	72 (44.2)	91 (55.8)	–2.4	2.4	
5-year survival status					**.018**
Alive	246 (55.4)	198 (44.6)	2.4	–2.4	
Dead	101 (45.7)	120 (54.3)	–2.4	2.4	
Synchronous metastases					**.007**
Metachronous	313 (54.2)	264 (45.8)	2.7	–2.7	
Synchronous	35 (38.9)	55 (61.1)	–2.7	2.7	
Disease recurrence					**.004**
No recurrence	255 (55.9)	201 (44.1)	2.8	–2.8	
Recurrence	93 (44.1)	118 (44.1)	–2.8	2.8	
T stage					**.002**
T1	38 (71.7)	15 (28.3)	3	–3	
T2	48 (60.8)	31 (39.2)	1.6	–1.6	
T3	173 (51.3)	164 (48.7)	–0.4	0.4	
T4	89 (45.2)	109 (54.8)	–2.4	2.4	
Nodal status					**.036**
Uninvolved	200 (54.9)	164 (45.1)	2.1	–2.1	
Involved	131 (46.6)	150 (53.4)	–2.1	2.1	
Metastasis					**.013**
Absent	312 (54.1)	265 (45.9)	2.5	–2.5	
Present	36 (40.0)	51 (60.0)	–2.5	2.5	
Perineural invasion					**.038**
Absent	275 (53.6)	238 (46.4)	2.1	–2.1	
Present	56 (43.4)	73 (56.6)	–2.1	2.1	
Tumor edge					**.01**
Infiltrative	132 (46.2)	154 (53.8)	–2.6	2.6	
Pushing	120 (58.0)	87 (42.0)	2.6	–2.6	

α-SMA, α–smooth muscle actin; pMMR, mismatch repair proficient.

^a^Adjusted residuals designate correlation strength in multiparameter analysis; significant *P* values highlighted in bold.

Similarly to the total cohort analyses, Kaplan-Meier survival analysis showed significant associations only between higher α-SMA stromal H-scores and CRC survival status in pMMR cases at the peripheral tumor compartment. Higher α-SMA at the peripheral tumor compartment in pMMR tumors was associated with poorer 3-year (*P* = .019) and 5-year (*P* = .015) survival FIGURE 2C, D. Following multivariate analysis with the covariates of T stage, nodal stage, metastasis, and grade, high α-SMA expression at the peripheral compartment in pMMR cases was found to be a significant independent prognostic indicator of 5-year survival (*P* = .044) TABLE 3.

**TABLE 3:** Multivariate Cox Regression Analysis of 5-Year Survival in Patients With Proficient Mismatch Repair Colorectal Cancer^a^

Clinicopathologic Variables	Significance	Exp (B)	95% CI for Exp (B)
Peripheral stromal H-score median	**.044**	1.323	1.008-1.738
T stage 1	**<.001**		
T stage 2	.603	.799	.344-1.860
T stage 3	.607	.824	.393-1.727
T stage 4	.264	1.539	.722-3.281
Nodal stage 0	**.017**		
Nodal stage 1	.103	1.316	.946-1.833
Nodal stage 2	**.005**	1.707	1.176-2.479
Metastasis	**<.001**	2.074	1.498-2.872
Grade 1	.462		
Grade 2	.697	1.257	.397-3.980
Grade 3	.402	1.705	.490-5.936

^a^Significant *P* values highlighted in bold.

Although some significant clinicopathologic correlations were also seen for α-SMA expression at luminal and central tumoral compartments (Supplementary Tables 6 and 7 respectively), no correlations were observed with survival.

### Deficient MMR Subgroup, Clinicopathologic Correlations, and Survival Analyses

There were no significant associations with clinicopathologic variables and average α-SMA stromal H-scores in dMMR tumors (Supplementary Table 8).

Kaplan-Meier survival analysis showed no significant associations between α-SMA stromal H-scores and CRC survival status in dMMR cases.

## DISCUSSION

As the molecular processes driving recurrence and metastases in CRCs are being understood, it is becoming evident that the tumor stroma plays a critical role in disease progression. However, other molecular determinants, such as MMR and CMS classification, also indicate differential prognosis.^14,15^ In this study, it is shown that a common available immunostain like α-SMA can be easily used to differentially prognosticate CRCs through simple stromal stain assessment, especially in pMMR cases. As manual assessment may be highly subjective, automated assessment on a digital platform was used to improve accuracy and eliminate subjectivity.

The role of SMA has been previously studied in various cancers and has been reported to be related to progression. Expression of α-SMA was widely observed in the stroma of invasive breast cancer with little expression in normal breast/fibroadenomas.^16^ In an earlier study in breast cancer, α-SMA expression was digitally quantified as the relative percentage within a preselected field area, and the high α-SMA group had a significantly poorer overall survival rate.^17^ Assessed by H-score, the presence of intratumoral CAFs expressing a high level of α-SMA correlated with poorer prognosis in luminal breast cancer.^18^ Assessed through immunofluorescence and digital spatial profiling, a high expression of α-SMA in the stromal compartment was associated with shorter disease-free survival and recurrence in early-stage HER2-positive breast cancers treated with trastuzumab.^19^ In lung adenocarcinomas, α-SMA positivity has been correlated with higher pTNM stages, including lymph node involvement.^20^ In pancreatic ductal adenocarcinomas, higher α-SMA immunostain (intensity and percentage) was significantly higher in tumors of a larger diameter (>3 cm), but no correlation was found with survival.^21^

In contrast, in CRC, α-SMA has been studied sparingly on clinical samples. In an early study of 192 CRCs, where MMR status was not investigated, greater α-SMA positivity within the stroma was associated with shorter disease-free survival but not with clinicopathologic variables.^12^ In another small series of 302 patients, tested for combinatorial stain analyses, the presence of high α-SMA expression and concomitant low podoplanin expression (assessed at the tumor center/invasive front together) was associated with the preoperative carcinoembryonic antigen (CEA) levels, tumor size, T stage and American Joint Committee on Cancer stage, infiltrative tumor border, high tumor budding, and microsatellite stable (MSS) tumors (*P* = .010). However, the microsatellite instable (MSI)/MSS status was available for only 146 of the 302 patients, and only 8 patients were MSI high.^11^ In the current study, α-SMA expression was evaluated holistically in the tumor stroma across all compartments and revealed wider associations with T stage, grade, vascular and perineural invasion, infiltrative tumor edge pattern, and proficient MMR status, revealing the importance of α-SMA assessment in CRC. Such associations were not observed in earlier series, where SMA was assessed on its own,^12^ probably indicating the advantages of testing a larger cohort. Whether in the whole cohort or in the selected pMMR subgroup, analysis in luminal, central, and peripheral compartments revealed significant associations with various adverse clinicopathologic parameters. Although there has been an early indication of a likely association with microsatellite stability status (a surrogate for MMR status) in a very small series, this earlier study^11^ did not offer any subgroup analyses based on MMR/MSI status, as it was likely underpowered in the MSI-high arm (only 8 patients). The current results show that the associations with poorer prognostic features are solely in the pMMR cohort, and hence, the case for utility of stromal α-SMA for prognostication is perhaps justified in the pMMR CRCs only. Within the consensus molecular classification of CRCs, pMMR tumors are heterogeneously distributed within CMS2-4,^8^ and within current clinical practice, there is limited scope of analyzing for CMS subtypes. However, a commonly used soft tissue stain in histopathology clinical departments such as α-SMA may provide a simple indirect indicator of adverse clinical features, including risk of nodal involvement, recurrence, and metastases, when assessed on digital platforms.

The association with pMMR status and significant associations in the subgroup parallel the morphology and perhaps reflect the underlying pathobiology of these cases. The dMMR tumors tend to be commonly more immunogenic, often with a mucinous or poorly differentiated or medullary-like morphology, with significant tumor-infiltrating lymphocytes,^22^ and therefore probably relatively lack α-SMA–positive CAFs in the microenvironment as compared to pMMR tumors. Some pMMR tumors show a prominent desmoplastic response and have the poorest prognosis among them (CMS4), with overactive mesenchymal signaling pathways.^23^ Indeed, the extracellular matrix produced by CAFs may limit the access of immune cells in terms of host immune response to the tumor and response to immunotherapy agents, which are therefore effective mainly for dMMR cases.^24-26^ The relative weakness of immune response and overactive mesenchymal signaling pathways in the α-SMA–overexpressing tumoral microenvironment may perhaps be one of the contributing factors to differential associations with adverse clinicopathologic variables and survival in pMMR cases. Other contributing factors may be the secretion of various chemokines, cytokines, and proteases by CAFs, including transforming growth factor β, vascular endothelial growth factor, IL-6, and matrix metalloproteinases, which promotes a fibrogenic milieu and potentiates local spread and distant metastases.^27,28^ As mesenchymal pathways are likely to be most active at the tumor periphery, it is intuitive that the expression of α-SMA in this compartment in pMMR tumors was found to be correlated with survival in the current study. The association with vascular invasion, perineural invasion, nodal spread, and recurrence in pMMR tumors reflects that high levels of stromal α-SMA may play a role in creating a microenvironment in pMMR CRCs that potentiates early and late locoregional spread and provides a primed milieu for later recurrence. Despite the association with recurrence/metastases, other studies have not found associations with patient survival. Even though in an earlier combinatorial study,^11^ low podoplanin and high α-SMA expression indicated a trend toward an association with poorer disease-free survival, it was not statistically significant, perhaps being underpowered in analysis. In another series of 289 patients with CRC, high mRNA expression levels of α‐SMA showed a trend toward shorter overall survival and a combination of CAF and M2 macrophage mRNA markers: α‐SMA, fibroblast‐specific protein 1, and fibroblast activation protein together predicted the outcome.^29^ These suggest that for improved survival prediction, perhaps combinatorial analysis with other markers may prove helpful. However, from the lessons learned from the current study, it may be worthwhile to carefully select patients by MMR status before proceeding to combinatorial analysis.

In conclusion, α-SMA immunostaining may have potential to indicate poorer prognostic features in pMMR CRCs but not in dMMR cases. As significant survival associations were observed for pMMR cases at the tumor periphery, selection for testing by pMMR status and assessing stain at the tumor periphery may prove beneficial. Stromal stain evaluation, in terms of intensity and percentage positivity, is challenging manually, and hence advances in digital pathology platforms may allow for easier scoring and detailed assessment of α-SMA, avoiding the subjectivity of manual estimation. Further functional and molecular studies with combinatorial biomarkers will help unveil the signaling pathways acting through the α-SMA active stroma within CRCs and help understand the complex pathobiology of the stromal microenvironment.

## Supplementary Material

aqae145_suppl_Supplementary_Tables_S1-8

## Data Availability

The data that support the findings of this study are available from the corresponding author upon reasonable request.

## Notes

Conflict of interest disclosure: The authors have nothing to disclose.
