# Supplementary material for: High α-SMA expression in the tumor stroma is associated with adverse clinical parameters in mismatch repair–proficient colorectal cancers only
Source: Am J Clin Pathol. 2024 Nov 4;163(3):464–72. doi: 10.1093/ajcp/aqae145 (PMC11890275; doi:10.1093/ajcp/aqae145)
Supplement: aqae145_suppl_Supplementary_Tables_S1-8 [file aqae145_suppl_supplementary_tables_s1-8.docx]

**Supplementary Table 1.** Complete tissue microarray clinical characteristics.

| **Clinicopathologic Features** | **No. of Cases (%)** |
| --- | --- |
| **Sex** |  |
| Male | 568 (56.8) |
| Female | 432 (43.2) |
| **Recurrence Status** |  |
| Recurrence | 713 (71.3) |
| No Recurrence | 287 (28.7) |
| **Tumor Location** |  |
| Right Colon | 363 (36.3) |
| Transverse Colon | 29 (2.9) |
| Left Colon | 363 (36.3) |
| Rectum | 147 (14.7) |
| **Final Stage** |  |
| Stage 1 | 161 (6.1) |
| Stage 2 | 402 (0.2) |
| Stage 3 | 319 (1.9) |
| Stage 4 | 118 (11.8) |
| **T Stage** |  |
| T1 | 74 (7.4) |
| T2 | 106 (10.6) |
| T3 | 526 (52.6) |
| T4 | 294 (29.4) |
| **Nodal Stage** |  |
| N0 | 570 (57.0) |
| N1 | 243 (24.3) |
| N2 | 161 (16.1) |
| **Metastasis** |  |
| Presence | 881 (88.1) |
| Absence | 119 (11.9) |
| **Tumor Grade** |  |
| Grade 1 | 20 (2) |
| Grade 2 | 886 (88.6) |
| Grade 3 | 93 (9.3) |
| **Vascular Invasion** |  |
| Absent | 502 (51.0) |
| Present | 483 (49.0) |
| **Perineural Invasion** |  |
| Absent | 783 (81.1) |
| Present | 183 (18.9) |
| **Lymphovascular Invasion** |  |
| Absent | 641 (69.1) |
| Present | 287 (30.9) |
| **MMR Status** |  |
| Proficient | 818 (83.6) |
| Deficient | 160 (16.4) |

**Supplementary Table 2.** χ² analysis for associations of the total cohorts average stromal α-SMA H-score with clinicopathologic variables.

| **Clinicopathologic Features** | **Average Stromal α-SMA H-Score** | | | | | **Adjusted Residuals** | | | | | **χ² *P*-value** |  |  |
| --- | --- | --- | --- | --- | --- | --- | --- | --- | --- | --- | --- | --- | --- |
|  | **Low (%)** | | | **High (%)** | | | **Low** | | **High** | |  |  |  |
| **Age** |  | | |  | | |  | |  | | .131 |  |  |
| Under 50 | | | 28 (41.2) | 40 (58.8) | | –1.5 | | | 1.5 | |  | | |
| Over 50 | | | 453 (50.7) | 441 (49.3) | | 1.5 | | | –1.5 | |  | | |
| **3-year Survival Status** | | |  |  | |  | | |  | | .203 | | |
| Alive | | | 374 (51.1) | 358 (48.9) | | 1.3 | | | –1.3 | |  | | |
| Dead | | | 105 (46.3) | 122 (53.7) | | –1.3 | | | 1.3 | |  | | |
| **5-year Survival Status** | | |  |  | |  | | |  | | .462 | | |
| Alive | | | 328 (50.8) | 318 (49.2) | | 0.7 | | | –0.7 | |  | | |
| Dead | | | 151 (48.2) | 162 (51.8) | | –0.7 | | | 0.7 | |  | | |
| **Synchronous Metastasis** | | |  |  | |  | | |  | | .005*** | | |
| Metachronous | | | 439 (51.6) | 411 (48.4) | | 2.8 | | | –2.8 | |  | | |
| Synchronous | | | 42 (37.5) | 70 (62.5) | | –2.8 | | | 2.8 | |  | | |
| **Disease Recurrence** | | |  |  | |  | | |  | | .000156*** | | |
| No Recurrence | | | 370 (53.9) | 317 (46.1) | | 3.8 | | | –3.8 | |  | | |
| Recurrence | | | 111 (40.4) | 164 (59.6) | | –3.8 | | | 3.8 | |  | | |
| **Tumor Site** | | |  |  | |  | | |  | | .010** | | |
| Right Colon | | | 240 (54.2) | 203 (45.8) | | 2.4 | | | –2.4 | |  | | |
| Left Colon | | | 149 (42.8) | 199 (57.2) | | –3.4 | | | 3.4 | |  | | |
| Rectum | | | 77 (54.2) | 65 (45.8) | | 1.1 | | | –1.1 | |  | | |
| Transverse Colon | | | 15 (51.7) | 14 (48.3) | | 0.2 | | | –0.2 | |  | | |
| **T-Stage** | | |  |  | |  | | |  | | .000324*** | | |
| T1 | | | 46 (65.7) | 24 (34.3) | | 2.7 | | | –2.7 | |  | | |
| T2 | | | 66 (64.1) | 37 (35.9) | | 3 | | | –3 | |  | | |
| T3 | | | 242 (47.5) | 268 (52.5) | | –1.7 | | | 1.7 | |  | | |
| T4 | | | 127 (45.5) | 152 (54.5) | | –1.8 | | | 1.8 | |  | | |
| **Nodal Stage** | | |  |  | |  | | |  | | .022* | | |
| N0 | | | 294 (53.3) | 258 (46.7) | | 2.7 | | | –2.7 | |  | | |
| N1 | | | 99 (43) | 131 (57) | | –2.3 | | | 2.3 | |  | | |
| N2 | | | 71 (46.1) | 83 (53.9) | | –0.9 | | | 0.9 | |  | | |
| **Nodal Status** | | |  |  | |  | | |  | | .007** | | |
| Uninvolved | | | 294 (53.3) | 258 (46.7) | | 2.7 | | | –2.7 | |  | | |
| Involved | | | 170 (44.3) | 214 (55.7) | | –2.7 | | | 2.7 | |  | | |
| **Metastasis** | | |  |  | |  | | |  | | .005** | | |
| Absent | | | 439 (51.6) | 411 (48.4) | | 2.8 | | | –2.8 | |  | | |
| Present | | | 42 (37.5) | 70 (62.5) | | –2.8 | | | 2.8 | |  | | |
| **Tumor Grade** | | |  |  | |  | | |  | | .002** | | |
| G1 | | | 8 (44.4) | 10 (55.6) | | –0.5 | | | 0.5 | |  | | |
| G2 | | | 414 (48.4) | 442 (51.6) | | –3 | | | 3 | |  | | |
| G3 | | | 59 (67.8) | 28 (32.2) | | 3.5 | | | –3.5 | |  | | |
| **Vascular Invasion** | | |  |  | |  | | |  | | .047* | | |
| Absent | | | 256 (52.8) | 229 (47.2) | | 2 | | | –2 | |  | | |
| Present | | | 214 (46.3) | 248 (53.7) | | –2 | | | 2 | |  | | |
| **Perineural Invasion** | | |  |  | |  | | |  | | .003** | | |
| Absent | | | 391 (52.1) | 360 (47.9) | | 3 | | | –3 | |  | | |
| Present | | | 70 (39.5) | 107 (60.5) | | –3 | | | 3 | |  | | |
| **Lymphovascular Invasion** | | |  |  | |  | | |  | | .733 | | |
| Absent | | | 307 (50.2) | 305 (49.8) | | 0.3 | | | –0.3 | |  | | |
| Present | | | 138 (48.9) | 144 (51.1) | | –0.3 | | | 0.3 | |  | | |
| **Tumor Edge** | | |  |  | |  | | |  | | 2.8301×10^–8^*** | | |
| Infiltrative | | | 149 (39.8) | 225 (60.2) | | –5.6 | | | 5.6 | |  | | |
| Pushing | | | 215 (60.4) | 141 (39.6) | | 5.6 | | | –5.6 | |  | | |
| **Tumor Budding** | | |  |  | |  | | |  | | .053 | | |
| Low | | | 256 (52.8) | 229 (47.2) | | 1.9 | | | –1.9 | |  | | |
| High | | | 106 (45.1) | 129 (54.9) | | –1.9 | | | 1.9 | |  | | |
| **Peritumoral Lymphocytes** | | |  |  | |  | | |  | | .413 | | |
| Inconspicuous | | | 258 (49) | 268 (51) | | –0.8 | | | 0.8 | |  | | |
| Conspicuous | | | 109 (52.4) | 99 (47.6) | | 0.8 | | | –0.8 | |  | | |
| **MMR Status** | | |  |  | |  | | |  | | 5.2223×10^–8^*** | | |
| Deficient | | | 366 (46) | 429 (54) | | 5.4 | | | –5.4 | |  | | |
| Proficient | | | 109 (69.9) | 47 (30.1) | | –5.4 | | | 5.4 | |  | | |

Adjusted residuals designate correlation strength in multiparameter analysis. Statistically significant values: **P* < .05; ***P* < .01; ****P* < .001.
H-score, histochemical score.

**Supplementary Table 3**. χ² analysis for associations of the total cohorts luminal stromal α-SMA H-scores with clinicopathologic variables.

| **Clinicopathologic Features** | **Luminal Stromal α-SMA H-Score** | | **Adjusted Residuals** | | **χ² *P*-value** |
| --- | --- | --- | --- | --- | --- |
|  | **Low (%)** | **High (%)** | **Low** | **High** |  |
| **Disease Recurrence** |  |  |  |  | .000169*** |
| No Recurrence | 347 (57.8) | 253 (42.2) | 3.8 | –3.8 |  |
| Recurrence | 102 (43.4) | 133 (56.6) | –3.8 | 3.8 |  |
| **Tumor Site** |  |  |  |  | .002** |
| Right Colon | 230 (59.3) | 158 (40.7) | 3 | –3 |  |
| Left Colon | 136 (45.3) | 164 (54.7) | –3.7 | 3.7 |  |
| Rectum | 66 (54.1) | 56 (45.9) | 0.1 | –0.1 |  |
| Transverse Colon | 17 (68) | 8 (32) | 1.4 | –1.4 |  |
| **T-stage** |  |  |  |  | .004** |
| T1 | 43 (67.2) | 21 (32.8) | 2.2 | –2.2 |  |
| T2 | 58 (66.7) | 29 (33.3) | 2.5 | –2.5 |  |
| T3 | 231 (51.8) | 215 (48.2) | –1.2 | 1.2 |  |
| T4 | 117 (49.2) | 121 (50.8) | –1.7 | 1.7 |  |
| **Nodal Stage** |  |  |  |  | .022* |
| N0 | 267 (55.6) | 213 (44.4) | 1.6 | –1.6 |  |
| N1 | 93 (45.1) | 113 (54.9) | –2.7 | 2.7 |  |
| N2 | 73 (57.9) | 53 (42.1) | 1.1 | –1.1 |  |
| **Tumor Grade** |  |  |  |  | .000804*** |
| G1 | 6 (42.9) | 8 (57.1) | –0.8 | 0.8 |  |
| G2 | 388 (51.9) | 359 (48.1) | –3.1 | 3.1 |  |
| G3 | 55 (74.3) | 19 (25.7) | 3.7 | –3.7 |  |
| **Vascular invasion** |  |  |  |  | .043* |
| Absent | 242 (56.8) | 184 (43.2) | 2 | –2 |  |
| Present | 197 (49.7) | 199 (50.3) | –2 | 2 |  |
| **Tumor edge** |  |  |  |  | .000020*** |
| Infiltrative | 139 (43) | 184 (57) | –4.3 | 4.3 |  |
| Pushing | 186 (60) | 124 (40) | 4.3 | –4.3 |  |
| **MMR status** |  |  |  |  | .000047*** |
| Deficient | 353 (50.7) | 343 (49.3) | 4.1 | –4.1 |  |
| Proficient | 93 (69.9) | 40 (30.1) | –4.1 | 4.1 |  |

Adjusted residuals designate correlation strength in multiparameter analysis. Statistically significant values: **P* < .05; ***P* < .01; ****P* < .001.
H-score, histochemical score; SMA, smooth muscle actin.

**Supplementary Table 4**. χ² analysis for associations of the total cohorts central stromal α-SMA H-scores with clinicopathologic variables.

| **Clinicopathologic Features** | **Central Stromal α-SMA H-Score** | | **Adjusted Residuals** | | **χ² *P*-value** |
| --- | --- | --- | --- | --- | --- |
|  | **Low (%)** | **High (%)** | **Low** | **High** |  |
| **T-Stage** |  |  |  |  | .032* |
| T1 | 39 (68.4) | 18 (31.6) | 2.7 | –2.7 |  |
| T2 | 44 (48.4) | 47 (51.6) | –0.5 | 0.5 |  |
| T3 | 223 (51.4) | 211 (48.6) | 0.3 | –0.3 |  |
| T4 | 116 (47) | 131 (53) | –1.5 | 1.5 |  |
| **Nodal Stage** |  |  |  |  | .029* |
| N0 | 256 (54.4) | 215 (45.6) | 2.6 | –2.6 |  |
| N1 | 87 (43.5) | 113 (56.5) | –2.3 | 2.3 |  |
| N2 | 65 (47.8) | 71 (52.2) | –0.7 | 0.7 |  |
| **Nodal Status** |  |  |  |  | .011* |
| Uninvolved | 256 (54.4) | 215 (45.6) | 2.6 | –2.6 |  |
| Involved | 152 (45.2) | 184 (54.8) | –2.6 | 2.6 |  |
| **Tumor Grade** |  |  |  |  | .004** |
| G1 | 8 (61.5) | 5 (38.5) | 0.8 | –0.8 |  |
| G2 | 361 (48.8) | 378 (51.2) | –3.3 | 3.3 |  |
| G3 | 52 (68.4) | 24 (31.6) | 3.2 | –3.2 |  |
| **Tumor Edge** |  |  |  |  | .000066*** |
| Infiltrative | 144 (43.4) | 184 (56.6) | –4 | 4 |  |
| Pushing | 180 (59.2) | 124 (40.8) | 4 | –4 |  |
| **Peritumoral Lymphocytes** |  |  |  |  | .02* |
| Inconspicuous | 224 (48.5) | 238 (51.5) | –2.3 | 2.3 |  |
| Conspicuous | 104 (58.8) | 73 (41.2) | 2.3 | –2.3 |  |
| **MMR Status** |  |  |  |  | .000004*** |
| Deficient | 327 (47.4) | 363 (52.6) | 4.6 | –4.6 |  |
| Proficient | 92 (69.2) | 41 (30.8) | –4.6 | 4.6 |  |

Adjusted residuals designate correlation strength in multiparameter analysis. Statistically significant values: **P* < .05; ***P* < .01; ****P* < .001.
H-score, histochemical score; SMA, smooth muscle actin.

**Supplementary Table 5.** χ² analysis for associations of the total cohorts peripheral stromal α-SMA H-scores with clinicopathologic variables.

| **Clinicopathologic Features** | **Peripheral Stromal α-SMA H-Score** | | **Adjusted Residuals** | | **χ² *P*-value** |
| --- | --- | --- | --- | --- | --- |
|  | **Low (%)** | **High (%)** | **Low** | **High** |  |
| **3-year Survival Status** |  |  |  |  | .013* |
| Alive | 326 (54.2) | 276 (45.8) | 2.5 | –2.5 |  |
| Dead | 87 (43.9) | 111 (56.1) | –2.5 | 2.5 |  |
| **5-year Survival Status** |  |  |  |  | .045* |
| Alive | 288 (54.1) | 244 (45.9) | 2 | –2 |  |
| Dead | 125 (46.6) | 143 (53.4) | –2 | 2 |  |
| **Synchronous Metastases** |  |  |  |  | .000206*** |
| Metachronous | 382 (54.1) | 324 (45.9) | 3.7 | –3.7 |  |
| Synchronous | 33 (34) | 64 (66) | –3.7 | 3.7 |  |
| **Disease Recurrence** |  |  |  |  | .003** |
| No Recurrence | 313 (55.1) | 255 (44.9) | 3 | –3 |  |
| Recurrence | 102 (43.4) | 133 (56.6) | –3 | 3 |  |
| **T-Stage** |  |  |  |  | .000324*** |
| T1 | 37 (66.1) | 19 (33.9) | 2.2 | –2.2 |  |
| T2 | 53 (61.6) | 33 (38.4) | 2 | –2 |  |
| T3 | 210 (49.8) | 212 (50.2) | –1.1 | 1.1 |  |
| T4 | 115 (48.1) | 124 (51.9) | –1.3 | 1.3 |  |
| **Metastasis** |  |  |  |  | .000473*** |
| Absent | 381 (54) | 325 (46) | 3.5 | –3.5 |  |
| Present | 34 (35.1) | 63 (64.9) | –3.5 | 3.5 |  |
| **Tumor Grade** |  |  |  |  | .038* |
| G1 | 8 (50) | 8 (50) | –0.5 | 0.5 |  |
| G2 | 360 (50.3) | 355 (49.7) | –2.3 | 2.3 |  |
| G3 | 47 (66.2) | 24 (33.8) | 2.6 | –2.6 |  |
| **Perineural Invasion** |  |  |  |  | .008** |
| Absent | 338 (53.6) | 293 (46.4) | 2.6 | –2.6 |  |
| Present | 60 (41.4) | 85 (58.6) | –2.6 | 2.6 |  |
| **Tumor Edge** |  |  |  |  | .002** |
| Infiltrative | 144 (45.4) | 173 (54.6) | –3.1 | 3.1 |  |
| Pushing | 165 (57.9) | 120 (42.1) | 3.1 | –3.1 |  |
| **MMR Status** |  |  |  |  | .000006*** |
| Deficient | 323 (48.4) | 344 (51.6) | 4.5 | –4.5 |  |
| Proficient | 90 (70.3) | 38 (29.7) | –4.5 | 4.5 |  |

Adjusted residuals designate correlation strength in multiparameter analysis. Statistically significant values: **P* < .05; ***P* < .01; ****P* < .001.
H-score, histochemical score; SMA, smooth muscle actin.

**Supplementary Table 6.** χ² analysis for associations of the pMMR cohorts luminal stromal α-SMA H-Score with clinicopathologic variables.

| **Clinicopathologic Features** | **pMMR Luminal Stromal α-SMA H-Score** | | **Adjusted Residuals** | | **χ² *P*-value** |
| --- | --- | --- | --- | --- | --- |
|  | **Low (%)** | **High (%)** | **Low** | **High** |  |
| **Disease Recurrence** |  |  |  |  | .003** |
| No Recurrence | 280 (58.5) | 199 (41.5) | 3 | –3 |  |
| Recurrence | 95 (46.1) | 111 (53.9) | –3 | 3 |  |
| **T-Stage** |  |  |  |  | .004** |
| T1 | 41 (68.3) | 19 (31.9) | 2.2 | –2.2 |  |
| T2 | 53 (68.8) | 24 (31.2) | 2.6 | –2.6 |  |
| T3 | 183 (51.7) | 171 (48.3) | –1.7 | 1.7 |  |
| T4 | 98 (50.5) | 96 (49.5) | –1.4 | 1.4 |  |
| **Vascular Invasion** |  |  |  |  | .037* |
| Absent | 196 (58.3) | 140 (41.7) | 2.1 | –2.1 |  |
| Present | 169 (50.3) | 167 (49.3) | –2.1 | 2.1 |  |
| **Perineural Invasion** |  |  |  |  | .023* |
| Absent | 295 (56.5) | 227 (43.5) | 2.3 | –2.3 |  |
| Present | 62 (45.6) | 74 (54.4) | –2.3 | 2.3 |  |
| **Tumor Edge** |  |  |  |  | .000167*** |
| Infiltrative | 127 (44.3) | 160 (55.7) | –3.8 | 3.8 |  |
| Pushing | 135 (61.1) | 86 (38.9) | 3.8 | –3.8 |  |

Adjusted residuals designate correlation strength in multiparameter analysis. Statistically significant values: **P* < .05; ***P* < .01; ****P* < .001.
H-score, histochemical score; pMMR, mismatch repair proficient; SMA, smooth muscle actin.

**Supplementary Table 7.** χ² analysis for associations of the pMMR cohorts central stromal α-SMA H-score with clinicopathologic variables.

| **Clinicopathologic Features** | **pMMR Central Stromal α-SMA H-Score** | | **Adjusted Residuals** | | **χ² *P*-value** |
| --- | --- | --- | --- | --- | --- |
|  | **Low (%)** | **High (%)** | **Low** | **High** |  |
| **T-Stage** |  |  |  |  | .011* |
| T1 | 38 (71.7) | 15 (28.3) | 3 | –3 |  |
| T2 | 44 (53) | 39 (47) | 0.3 | –0.3 |  |
| T3 | 176 (51.6) | 165 (48.4) | 0 | 0 |  |
| T4 | 93 (46) | 109 (54) | –1.9 | 1.9 |  |
| **Nodal Stage** |  |  |  |  | .045* |
| N0 | 201 (55.5) | 161 (44.5) | 2.5 | –2.5 |  |
| N1 | 82 (45.8) | 97 (54.2) | –1.7 | 1.7 |  |
| N2 | 53 (45.7) | 63 (54.3) | –1.3 | 1.3 |  |
| **Nodal Status** |  |  |  |  | .013* |
| Uninvolved | 201 (55.5) | 161 (44.5) | 2.5 | –2.5 |  |
| Involved | 135 (45.8) | 160 (54.2) | –2.5 | 2.5 |  |
| **Tumor Edge** |  |  |  |  | .000033*** |
| Infiltrative | 125 (42.5) | 169 (57.5) | –4.1 | 4.1 |  |
| Pushing | 132 (61.1) | 84 (38.9) | 4.1 | –4.1 |  |

Adjusted residuals designate correlation strength in multiparameter analysis. Statistically significant values: **P* < .05; ***P* < .01; ****P* < .001.
H-score, histochemical score; pMMR, mismatch repair proficient; SMA, smooth muscle actin.

**Supplementary Table 8.** χ² analysis for associations of the dMMR cohorts average stromal α-SMA H-Score with clinicopathologic variables.

| **Clinicopathologic Features** | **dMMR Average Stromal α-SMA H-Score** | | **Adjusted Residuals** | | **χ² *P*-value** |
| --- | --- | --- | --- | --- | --- |
|  | **Low (%)** | **High (%)** | **Low** | **High** |  |
| **Age** |  |  |  |  | .069 |
| Under 50 | 2 (22.2) | 7 (77.8) | –1.8 | 1.8 |  |
| Over 50 | 77 (53.5) | 67 (46.5) | 1.8 | –1.8 |  |
| **3-year Survival Status** |  |  |  |  | .460 |
| Alive | 56 (49.6) | 57 (50.4) | –0.7 | 0.7 |  |
| Dead | 22 (56.4) | 17 (43.6) | 0.7 | –0.7 |  |
| **5-year Survival Status** |  |  |  |  | .94 |
| Alive | 50 (51.5) | 47 (48.5) | 0.1 | –0.1 |  |
| Dead | 28 (50.9) | 27 (49.1) | –0.1 | 0.1 |  |
| **Synchronous Metastasis** |  |  |  |  | .656 |
| Metachronous | 75 (52.1) | 69 (47.9) | 0.4 | –0.4 |  |
| Synchronous | 4 (44.4) | 5 (55.6) | –0.4 | 0.4 |  |
| **Disease Recurrence** |  |  |  |  | .848 |
| No Recurrence | 65 (52) | 60 (48) | 0.2 | –0.2 |  |
| Recurrence | 14 (50) | 14 (50) | –0.2 | 0.2 |  |
| **Tumor Site** |  |  |  |  | .175 |
| Right Colon | 65 (51.2) | 62 (48.8) | –0.2 | 0.2 |  |
| Left Colon | 11 (52.4) | 10 (47.6) | 0.1 | –0.1 |  |
| Rectum | 3 (100) | 0 (0) | 1.7 | –1.7 |  |
| Transverse Colon | 0 (0) | 2 (100) | –1.5 | 1.5 |  |
| **T-Stage** |  |  |  |  | .97 |
| T1 | 3 (60) | 2 (40) | 0.4 | –0.4 |  |
| T2 | 4 (57.1) | 3 (42.9) | 0.3 | –0.3 |  |
| T3 | 49 (51) | 47 (49) | –0.2 | 0.2 |  |
| T4 | 23 (51.1) | 22 (48.9) | –0.1 | 0.1 |  |
| **Nodal Stage** |  |  |  |  | .418 |
| N0 | 51 (48.1) | 55 (51.9) | –1.3 | 1.3 |  |
| N1 | 14 (58.3) | 10 (41.7) | 0.7 | –0.7 |  |
| N2 | 14 (60.9) | 9 (39.1) | 1 | –1 |  |
| **Nodal Status** |  |  |  |  | .191 |
| Uninvolved | 51 (48.1) | 55 (51.9) | –1.3 | 1.3 |  |
| Involved | 28 (59.6) | 19 (40.4) | 1.3 | –1.3 |  |
| **Metastasis** |  |  |  |  | .656 |
| Absent | 75 (52.1) | 69 (47.9) | 0.4 | –0.4 |  |
| Present | 4 (44.4) | 5 (55.6) | –0.4 | 0.4 |  |
| **Tumor Grade** |  |  |  |  | .073 |
| G1 | 1 (33.3) | 2 (66.7) | –0.6 | 0.6 |  |
| G2 | 49 (46.2) | 57 (53.8) | –2 | 2 |  |
| G3 | 29 (65.9) | 15 (34.1) | 2.2 | –2.2 |  |
| **Vascular Invasion** |  |  |  |  | .189 |
| Absent | 43 (47.3) | 48 (52.7) | –1.3 | 1.3 |  |
| Present | 36 (58.1) | 26 (41.9) | 1.3 | –1.3 |  |
| **Perineural Invasion** |  |  |  |  | .866 |
| Absent | 67 (51.9) | 62 (48.1) | 0.2 | –0.2 |  |
| Present | 11 (50) | 11 (50) | –0.2 | 0.2 |  |
| **Lymphovascular Invasion** |  |  |  |  | .181 |
| Absent | 50 (49) | 52 (51) | –1.3 | 1.3 |  |
| Present | 28 (60.9) | 18 (39.1) | 1.3 | –1.3 |  |
| **Tumor Edge** |  |  |  |  | .893 |
| Infiltrative | 20 (54.1) | 17 (45.9) | 0.1 | –0.1 |  |
| Pushing | 48 (52.7) | 43 (47.3) | –0.1 | 0.1 |  |
| **Tumor Budding** |  |  |  |  | .429 |
| Low | 46 (52.9) | 41 (47.1) | –0.8 | 0.8 |  |
| High | 23 (60.5) | 15 (39.5) | 0.8 | –0.8 |  |
| **Peritumoral Lymphocytes** |  |  |  |  | .684 |
| Inconspicuous | 40 (51.9) | 37 (48.1) | –0.4 | 0.4 |  |
| Conspicuous | 30 (55.6) | 24 (44.4) | 0.4 | –0.4 |  |

Adjusted residuals designate correlation strength in multiparameter analysis. Statistically significant values: **P* < .05; ***P* < .01; ****P* < .001.
H-score, histochemical score; dMMR, mismatch repair deficient; SMA, smooth muscle actin.
